# Isolation and Molecular Identification of *Acanthamoeba* and *Naegleria* from Agricultural Water Canal in Qazvin, Iran

**DOI:** 10.18502/ijpa.v15i3.4204

**Published:** 2020

**Authors:** Mandana KHORSANDI RAFSANJANI, Elham HAJIALILO, Mehrzad SARAEI, Safar Ali ALIZADEH, Amir JAVADI

**Affiliations:** 1. Student Research Committee, Qazvin University of Medical Sciences, Qazvin, Iran; 2. Cellular and Molecular Research Center, Research Institute for Prevention of Non-Communicable Diseases, Qazvin University of Medical Sciences, Qazvin, Iran; 3. Department of Parasitology and Mycology, Qazvin University of Medical Sciences, Qazvin, Iran; 4. Medical Microbiology Research Center, Qazvin University of Medical Sciences, Qazvin, Iran; 5. Department of Microbiology, Qazvin University of Medical Sciences, Qazvin, Iran; 6. Department of Social Sciences, School of Medicine, Qazvin University of Medical Sciences, Qazvin, Iran

**Keywords:** *Acanthamoeba*, *Naegleria*, Genotype, Agricultural water, Iran

## Abstract

**Background:**

Free-living amoeba (FLA) are widely distributed in different environmental sources. The most genera of the amoeba are *Acanthamoeba*, *Naegleria* and *Vermamoeba.* The most common consequences of the infections in immune-deficient and immuno-competent persons are amoebic encephalitis and keratitis. The aim of this study was to investigate the presence of *Acanthamoeba* spp. and *Naegleria* spp., isolated from the main agricultural water canal in Qazvin.

**Methods:**

Totally, 120 water specimens were collected and later the specimens were cultured and cloned to identify positive samples. PCR amplification and sequencing were carried out to identify the isolated species as well as the genotypes of amoeba.

**Results:**

According to morphological surveys, 41.7% (50/120) of water specimens were positive for FLA. Molecular analysis revealed that 68.6% and 31.4% of *Acanthamoeba* specimens were identified as T3 and T4 genotypes, respectively. Also, two species of *Naegleria* named as *N. lovaniensis* (57.1%) and *Naegleria* sp. (42.8%) were identified. The results of pathogenicity assays demonstrated that 38.5% of T3 and 61.5% of T4 genotypes of *Acanthamoeba* were highly pathogenic parasites.

**Conclusion:**

The water flowing in the agricultural canal of the area is contaminated with potential pathogenic FLA, therefore, it is recommended that more attention to be paid towards proper treatment of water sources to prevent possible risk of the disease.

## Introduction

Free-living amoeba (FLA) are ubiquitous protozoan, widely distributed in nature consisting of several common genera such as *Acanthamoeba*, *Naegleria, Vermamoeba* and *Balamuthia.* These microorganisms have been called amphizoic amoeba with pathogenic potential in humans and animals (1, 2). Diversified species of the amoeba are present in a wide array of habitats globally, including fresh and sea water, hot tubs, mineral springs, swimming pools, soil and dust, ventilators, and dialysis machines (2–4). This protozoan parasite has a life cycle consisting of active trophozoite stage and pleomorphic cyst stage, with high tolerance against many harsh environments and toxic substances (2, 3).

In spite of widespread environmental distribution of *Acanthamoeba* spp., the disease incidence is relatively low which is particularly due to insufficient susceptible hosts, i.e. contact lens consumers and immunocompromised individuals (AIDS and diabetic patients, tissue graft recipients, pregnant women as well as persons undergoing corticosteroid therapy) (5). However, *Acanthamoeba* spp. can elicit several harsh clinical sequelae such as granulomatous amoebic encephalitis (GAE), skin lesions and nasopharyngeal infections particularly in individuals with suppressed immune status (3). On the contrary, *Acanthamoeba* keratitis (AK) patients possess efficient immunity and such ocular complication occurs via using unclean contact lenses, swimming in contaminated water supplies and corneal abrasion owing to exogenous agents (3, 6). In addition to these concerns, *Acanthamoeba* spp. are considered as potent reservoirs of pathogenic microorganisms, which would support their evasion from immune system, replication, and transmission to susceptible hosts (2, 3).

*Naegleria* spp. are free-living amoeboflagel-late protists mostly prevalent during warm seasons in both fresh and warm waters so that exposure to environmental water sources may cause the development of infection. This parasite causes a highly lethal, fulminant disease called primary amebic meningoencephalitis (PAM) (7, 8). While rare, the importance of infections with FLA in humans emphasizes more comprehensive studies on the prevalence of such opportunistic organisms in our surroundings. Based on 18S rRNA gene sequence, 22 *Acanthamoeba* genotypes (T1–T22) have been discerned from environmental sources and clinical cases (http://u.osu.edu/acanthamoeba/genomes-of-acanthamoeba) (9–12). T4 is the most frequent genotype isolated from environmental samples and AK patients globally, implicating more pathogenic capability of this genotype (3, 13, 14). The only clinical disease observed in Iran is the AK due to the T2, T3, T4, T9, and T11 genotypes (14–16). On the other hand, *Naegleria* sp. has been isolated from several regions of the country (17–20).

We aimed to isolate and identify of waterborne FLA belonging to *Acanthamoeba* spp. and *Naegleria* spp. from the water flowing in the major water canal used for agricultural purposes in Qazvin, north-west of Iran using morphological and molecular analysis.

## Materials and Methods

### Study area and sampling procedure

**Fig. 1:**
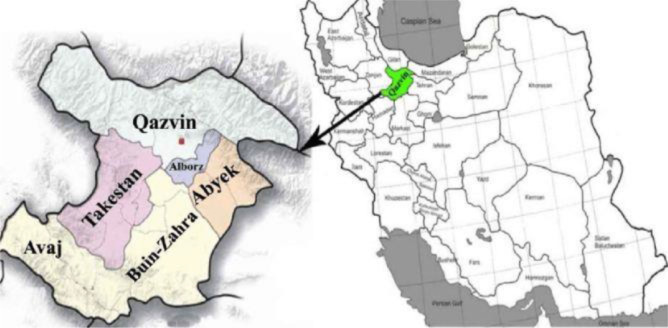
Map of Qazvin province. It is located in northern margin of central Iran

This was a cross-sectional study performed during spring and summer seasons of 2018 (from Apr to Aug) in Qazvin Province, located in the northern margin of central Iran (Fig. 1).

Sampling was done monthly by random collecting of water specimens in sterile 500 ml containers from the main agricultural waterway. Samples were collected three-times per month with 10-day time intervals, each time with 10 bottles of water samples, yielding a number of 30 water samples per month and 120 bottles totally. Water temperature (14–17 °C) and pH (7–8) of sampling areas were recorded.

Fisher’s and chi-square tests were used to determine statistics analysis. *P* value <0.05 was considered as significant. All specimens were examined at parasitology laboratory of Qazvin Medical School.

### Water filtration, amoeba culture and harvesting

To isolate FLA, water samples were passed through a nitrocellulose filter membrane (pore size: 0.45 μm) using a vacuum pump; so that the amoeba remained on the filter surface and not going through the sieve. Then, filters were inversely placed on the surface of 1.5% non-nutrient agar (NNA) plate seeded with *Escherichia coli* bacteria and incubated at 25–30 °C (16, 21). Cultures were followed up, up to 21 days in order to find trophozoites and/or cysts of the amoeba. Positive isolates were cloned to achieve a plate without bacterial and fungal contamination.

### Acanthamoeba Pathogenicity tests

Osmo-tolerance and thermo-tolerance assays were used for pathogenicity surveys. The growth ability of *Acanthamoeba* in two concentrations of 0.5 M and 1 M mannitol in a non-nutrient agar and under 37 °C and 40 °C were examined in Osmo-tolerance and thermo-tolerance assays, respectively. The specimens were followed up daily for a duration of one week (14, 22, 23).

### DNA extraction and PCR amplification

The amoeba were collected in sterile phosphate buffered saline (PBS) from positive culture plates. DNA extraction was performed by High Pure polymerase chain reaction (PCR) Template.

The preparation kit (Roche, Mannheim, Germany) was used according to manufacturer’s protocol along with glass beads treatment (14). PCR optimization was performed using *Acanthamoeba* specific primers of JDP1 5′- GGCCCAGATCGTTTACCGTGAA-3′ and JDP2 5′- TCTCACAAGCTGCTAGGGAGTCA-3′ to amplify an approximately the 500 bp length fragment within the 18S rRNA gene region, Also, *Naegleria* specific primers NA1 5′-AACCTGCGTAGGGATCAT-3′ and NA2 5′- TTTTCTTTTCCTCCCCTTAT -3′ amplified an approximately 400bp piece. Standard PCR for both amoeba were done in a total volume of 30 μl containing ready-made mixture of Amplicon (Taq DNA Polymerase Master Mix RED, Denmark), template DNA, 0.1 μM of each primer. Moreover, a negative control was considered for each amplification process. The thermal cycler conditions for *Acanthamoeba* were as follows: an initial denaturation step at 94 °C for 4 min; 30 cycles of denaturation at 94 °C for 30 sec, annealing at 64 °C for 45 sec and extension at 72 °C for 45 sec; and a final extension step at 72 °C for 7 min. Furthermore, DNA amplification protocol for *Naegleria* included: a primary denaturation step at 95 °C for 5 min; 30 cycles of denaturation at 95 °C for 20 sec, annealing at 57 °C for 20 sec and extension at 72 °C for 30 sec; and a final extension step at 72 °C for 5 min. Finally, the PCR products were electrophoresed on agarose gel (2% w/v), followed by observing the appeared bands under ultraviolet illumination.

### DNA Sequencing

To identify the genotype of amoeba, after purification, the positive specimens were sequenced by ABI3130 sequencer machine (Applied Biosystems, USA). The data obtained in sequencing were edited manually by chromas (Version 1.0.0.1) software, and further compared by aligning the query sequence against the eukaryotic sequences using BLAST program (https://blast.ncbi.nlm.nih.gov/Blast.cgi) on GenBank nucleotide sequence database, resulting in genotype determination. The sequences were deposited in the GenBank database under the Accession Nos. MK347298-MK347321, MK347323-MK347328, and MK347330-MK347341.

### Ethical approval

The procedure implemented in the present research project was fully reviewed and approved by the Research Ethics Committee of Qazvin University of Medical Sciences (Code no: IR.QUMS.REC.1396.183).

## Results

**Fig. 2:**
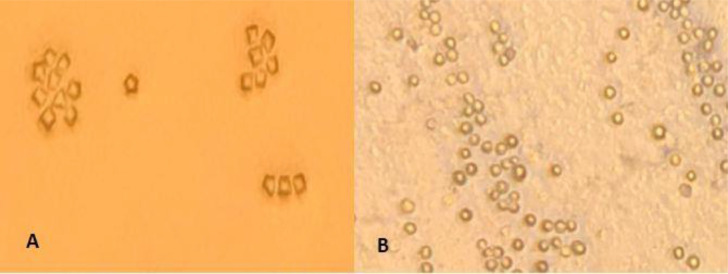
Light microscopy photographs of (A) *Acanthamoeba* and (B) Vahlkampfiids cysts (magnification X100)

In total, 50 out of 120 (41.7%) water samples cultured in NNA were positive for FLA, among which 35 (29.2%) and 11 (9.2%) were *Acanthamoeba* and Vahlkampfiids amoebae, respectively., In addition, 4 (3.3%) of samples were identified as mixed contamination in microscopic surveys (Fig. 2) (Table 1).

**Table 1:** Frequency of isolated *Acanthamoeba* and Vahlkampfiids in Qazvin agricultural water canal, by culture and microscopy

***Season***	***Acanthamoeba N (%)***	***Vahlkampfiids N (%)***	***Acanthamoeba & Vahlkampfiids N (%)***	***Total N (%)***
Spring	18 (72)	4 (16)	3 (12)	25 (50)
Summer	17 (68)	7 (28)	1 (4)	25 (50)
Total	35 (70)	11 (22)	4 (8)	50 (100)

The frequency of isolated FLAs from agricultural water canal was similar in two warm seasons, however the *Naegleria* species was more common in summer rather than spring although the statistical analysis failed to demonstrate any significant correlation between the type of parasite and their prevalence in two different seasons. All *Acanthamoeba* species in cultures were confirmed by molecular test, while 63.6% (7/11) of the Vahlkampfiids amoebae identified in cultures, were detected as *Naegleria* species by PCR. Sequencing output determined that 68.6% (24/35) of *Acanthamoeba* isolates were T3 genotype, whereas 31.4% (11/35) of the isolates were dedicated to T4 genotype. Of seven *Naegleria* isolates, four (57.1%) belonged to *N. lovaniensis* and three (42.8%) were identified as *Naegleria* sp. Among 35 *Acanthamoeba* isolates 13 (37%) were considered as highly pathogenic, among those 38.5% (5/13) were T3 genotype and 61.5% (8/13) T4 genotype (Table 2).

**Table 2:** Data obtained for *Acanthamoeba* collected from agricultural water canal samples in Qazvin province, Iran

**Isolate code**	**Genotype**	**Accession number**	**Osmo tolerant**		**Termo tolerant**	

			**0.5 M**	**1 M**	**37 °c**	**42 °c**
QAS-K2	T3	MK347298	+	−	+	−
QAS-K11	T3	MK347299	−	−	−	−
QAS-K17	T3	MK347300	−	−	−	−
QAS-K23	T3	MK347301	−	−	−	−
QAS-K28	T3	MK347302	+	−	+	−
QAS-K38	T3	MK347303	+	−	+	−
QAS-K39	T3	MK347304	−	−	−	−
QAS-K41	T3	MK347305	+	−	+	−
QAS-K42	T3	MK347306	+	−	+	−
QAS-K48	T3	MK347307	−	−	−	−
QAS-K50	T3	MK347308	+	−	+	−
QAS-K53	T3	MK347309	+	+	+	+
QAS-K54	T3	MK347310	+	−	+	−
QAS-K56	T3	MK347311	+	−	+	−
QAS-K65	T3	MK347312	+	+	+	+
QAS-K71	T3	MK347313	+	−	+	−
QAS-K79	T3	MK347314	+	−	+	−
QAS-K84	T3	MK347315	+	−	+	−
QAS-K88	T3	MK347316	+	+	+	+
QAS-K97	T3	MK347317	−	−	−	−
QAS-K109	T3	MK347318	+	+	+	+
QAS-K111	T3	MK347319	+	−	+	−
QAS-K114	T3	MK347320	+	+	+	+
QAS-K95	T3	MK347321	+	−	+	−
QAS-K73	T4	MK347323	+	−	+	−
QAS-K8	T4	MK347324	+	−	+	−
QAS-K10	T4	MK347325	+	+	+	+
QAS-K15	T4	MK347326	+	+	+	+
QAS-K18	T4	MK347327	+	+	+	+
QAS-K29	T4	MK347328	+	+	+	+
QAS-K75	T4	MK347330	+	+	+	+
QAS-K81	T4	MK347331	−	−	−	−
QAS-K101	T4	MK347332	+	+	+	+
QAS-K30	T4	MK347333	+	+	+	+
QAS-K104	T4	MK347334	+	+	+	+

## Discussion

Our knowledge on the FLAs and the risk of human infections are still disputed. The geoecological distributions of such amoebas and their putative genotypes have been interesting fields of study during recent years (24–26, 21). Herein, we have evaluated the frequency and genotypes of *Acanthamoeba* and *Naegleria* spp. in agricultural water canal of Qazvin province, which irrigates all agricultural lands of the province. Based on our findings, of 120 water samples 41.7% were positive for FLAs, which contributes to half of the reported prevalence in Qazvin stagnant waters (26). Such difference could be related to the difference in the number of samples used in both studies. Only two studies were previously done on the prevalence of FLAs in agricultural water canal; the first was from Ahvaz, in which the authors reported a prevalence of 71.6% for all water sources including agricultural canal (27) and the second was from Bojnourd, reporting a prevalence of 57.14% in agricultural canal water (28). There exist several investigations on the prevalence of FLAs in various water sources across the country and around the globe, indicating the diversity and distribution of such amoeba. For instance, 59.1% of 22 cultured surface and stagnant water samples in Tehran (29), 35% of 120 water samples (wells and water pipes) in Shiraz (30), 61.11% of 54 water samples (wells, springs, qanats, and motor houses) in Arak (31), 30% of 50 environmental water sources (sea, pools, ponds, fountains, and running waters) in various regions of Guilan province (32), 88% of 93 water specimens from pools and ponds in Sistan & Baluchistan (33), and 34.44% of 90 pond water samples in Mashhad contaminated with the amoeba (34). Furthermore, contamination of water sources with FLA has been documented in reports from Egypt (43.2%), Italy (28.7%) (35, 36), Thailand (15.9%), China (14.68%), Japan (68.7%), Turkey (22%), and Hungary (41%) (37–41).

The water quality used for agricultural purposes is influenced primarily by salinity and water hardness. It has been proved that *Acanthamoeba* cysts are very resistant to several harsh environments such as excessive chlorine concentrations in water (3).

Based on our findings, 3.3% (4/120) of water samples from agricultural canal were contaminated with *Naegleria lovaniensis*. Although, this species is a non-pathogenic organism in human, yet it can grow at 45 °C similar to *N. fowleri*; this issue implicates the simultaneous presence of both species in the environment (42). The frequency of *Naegleria* is low in Iran, which signifies far less prevalence of this amoeba than *Acanthamoeba*. A prevalence of 15% was shown for *Naegleria* in rivers and ponds of the city of Rasht (20). Also, 26.7% of 30 water samples from Ardabil hot tubs were contaminated (18). Similar contaminations were documented in reports from Mashhad and Semnan (43, 44).

In Turkey, *Naegleria* spp. was also found in much lower prevalence than *Acanthamoeba* (45). However, it is also claim that 92.9% of 70 environmental water specimens from parks in China were contaminated to *Naegleria* (46).

In the current study, only T3 and T4 genotypes of *Acanthamoeba* were identified among the specimens, although T4 is reported to be the predominant genotype in water and clinical samples, yet the prevalence of T3 (68.6%) genotype was higher than T4 (31.4%) genotype in our present study (3, 13, 14). Consistent with our results, T3 genotype was dominant in a research from Osaka, Japan (39). According to pile of research in different parts of Iran T2–T3, T11, T13, and T15 genotypes of *Acanthamoeba* were found in various water sources of the country (27, 47–52). In this study, 37% of isolates were considered as highly pathogenic, in which the number of T4 genotype was higher than T3 genotype. Our results indicated that T4 genotype possessed more pathogenicity, compared to T3 genotype, which is consistent with previous research (49). The current investigation is the first molecular evaluation of FLA in agricultural water canal in this area of the country. Our results confirm the presence of *N. lovaniensis* as well as pathogenic T3 and T4 genotypes of *Acanthamoeba* in the agricultural water resource of the province.

Hence, it is recommended that more attention is needed on better supervising the implementation of health standards for water sources specifically agricultural water to prevent the occurrence of any unpleasant event in the public health of the society.

## Conclusion

T3, and T4 genotypes of *Acanthamoeba* also *Naegleria* species were found in the agricultural water of the study area with the potential to threat the general health of the community. The findings of this study highlight the necessity for microbiological examinations of water sources, to prevent especially the young individuals from contracting diseases when swimming in such waters.
